# Comparative Elemental Profiling and Differentiation Analysis of *Lonicerae japonicae* Flos and *Lonicerae* Flos Based on ICP-MS/AES and Chemometrics

**DOI:** 10.3390/ijms27167276

**Published:** 2026-08-14

**Authors:** Chao Yu, Yanxia Shu, Xiuli Li, Zongshuo Li, Fan Su, Meng Sun, Zhenghui Liu, Weidong Li

**Affiliations:** 1 School of Chinese Materia Medica, Beijing University of Chinese Medicine, Beijing 102488, China; yuchao202111@163.com (C.Y.); shuyanxia66@163.com (Y.S.); 2 Collaborative Innovation Center of Aerospace and Traditional Chinese Medicine, Beijing University of Chinese Medicine, Beijing 102488, China; 3 Institute of Agricultural Product Quality Safety and Nutrition, Tianjin Academy of Agricultural Sciences, Tianjin 300381, China; lixiuli@taas.ac.cn (X.L.); sufan@taas.ac.cn (F.S.); axsuar@163.com (M.S.); 4 School of Life Sciences, Beijing University of Chinese Medicine, Beijing 102488, China; lizongshuo2009@126.com

**Keywords:** inorganic elements, Fe/Mn ratio, *Lonicerae japonicae* flos, *Lonicerae* flos, network pharmacology, molecular docking

## Abstract

*Lonicerae japonicae* flos (LJ), derived from *Lonicera japonica* Thunb., is a classic medicinal and edible plant traditionally used to clear heat, detoxify, and disperse wind-heat. It is widely consumed as a health tea or food additive and played a significant role during the COVID-19 pandemic. Its closely related species, *Lonicerae* flos (LFS), derived from *L*. *macranthoides*, *L. hypoglauca*, *L. confusa*, and *L. fulvotomentosa*, share similar functions. Distinguishing between these two is crucial to minimizing medication-related risks; however, studies on their differences in inorganic elements are currently insufficient. This study investigated the content differences in ten inorganic elements in 97 samples using inductively coupled plasma mass spectrometry and inductively coupled plasma atomic emission spectrometry. LJ exhibited significantly higher iron (Fe) content and considerably lower manganese (Mn) content than LFS. Fe, Mn and Na were identified as key discriminatory elements through orthogonal partial least squares-discriminant analysis, and cluster analysis based on the Fe/Mn ratio also achieved clear separation between the two groups. Network pharmacology and pathway enrichment analyses were subsequently conducted to explore the potential biological relevance of Fe- and Mn-associated differences. Additionally, molecular docking provided exploratory computational assessments of theoretical interactions between Fe/Mn and selected target proteins under simplified conditions. It is important to note that these computational results should not be interpreted as direct evidence of biological activity, since no in vitro or in vivo validation was performed. Overall, this study provides elemental profiling data to differentiate between LJ and LFS and highlights the potential value of the Fe/Mn ratio as a complementary chemical indicator for the authentication and quality evaluation of *Lonicera* medicinal materials.

## 1. Introduction

*Lonicerae japonicae* flos (LJ), derived from the dried flower buds or newly bloomed flowers of *Lonicera japonica* Thunb., has been used medicinally in China for centuries [1]. As a well-recognized medicinal and edible plant, it is also widely consumed as a health tea and used as a food ingredient in various products, such as beverages and confectioneries [2,3,4,5]. Owing to its traditional functions of clearing heat and detoxifying, LJ and its related preparations were extensively used in the prevention and management of the COVID-19 pandemic, further highlighting its significant medicinal value and application potential [6]. The closely related species, *Lonicerae* flos [LFS, derived from *L. macranthoides* (LM), *L. hypoglauca* (LH), *L. confusa* (LC), and *L. fulvotomentosa* (LF)], share comparable traditional functions. However, their material basis and biological activities may differ substantially [7]. Using the two interchangeably in practical application may compromise therapeutic efficacy and medication safety. To clarify these differences, previous comparative studies have largely focused on organic constituents such as organic acids, flavonoids, and saponins [8,9,10,11], whereas investigations on inorganic elements remain scarce.

In recent years, the increasing emphasis on quality control in traditional Chinese medicine (TCM) has gradually unveiled the importance of inorganic elements as key determinants of the intrinsic characteristics of medicinal materials, serving as potential markers for both provenance and quality [12]. Research has demonstrated that inorganic elements in TCM formulations also possess pharmacological activity and are considered integral to the material basis of efficacy, playing synergistic or antagonistic roles in therapeutic effects [13]. Notably, the relative abundance of iron (Fe) and manganese (Mn) has been reported to be associated with the therapeutic efficacy of traditional Chinese medicines, with the Fe/Mn ratio increasingly recognized as a discriminative marker among them [14,15]. However, large-scale quantitative comparisons of complete elemental profiles between LJ and LFS are still lacking, which limits the identification of characteristic differential elements. Furthermore, previous studies on elemental composition have primarily focused on content determination and classification modeling, with few incorporating bioinformatic analyses to formulate testable hypotheses regarding the potential biological relevance of differential trace elements.

Network pharmacology, originally developed for the analysis of organic small-molecule active ingredients, has recently been expanded to investigate trace element regulatory networks, particularly in studies focusing on metal homeostasis [16,17]. This workflow is well-suited for elemental datasets, as it can systematically map multi-target biological networks of Fe and Mn based on existing database annotations of metal transport and binding proteins. This process facilitates the generation of testable research hypotheses for follow-up functional verification. Molecular docking serves as an auxiliary in silico tool to network pharmacology [18]; however, it is important to clarify that simulations using AutoDock Vina, which rely on default force fields, cannot provide direct, definitive molecular evidence of physiological protein-ion interactions. Instead, they only yield simplified predictions of static binding tendencies in a vacuum environment. Consequently, this study distinguishes between the analytical and biological dimensions of elemental research. The primary objective is to characterize and compare the elemental profiles of LJ and LFS, as well as to assess their potential utility in species discrimination and quality evaluation. Discussions regarding the biological implications of the differences in Fe/Mn are limited to exploratory computational inferences and remain to validated through dedicated experimental studies. To our knowledge, few studies have successfully integrated quantitative elemental analysis, chemometric discrimination, and element-oriented network pharmacology within a unified framework to comparatively investigate LJ and LFS.

In this context, the present study was conducted to quantitatively assess ten inorganic elements (Fe, Mn, Cu, Zn, Na, Cr, Mo, Ni, As, and Cd) in 97 batches of LJ and LFS samples using ICP-MS and ICP-AES. The aim was to identify differential elements and evaluate the discriminatory potential of the Fe/Mn ratio through OPLS-DA and cluster analyses. Additionally, network pharmacology and molecular docking were utilized as exploratory computational methods to formulate testable hypotheses concerning the biological significance of the differential accumulation of Fe and Mn. The findings provide elemental fingerprint data for distinguishing between LJ and LFS and establish a foundation for future experimental research into their material basis differences.

## 2. Results

### 2.1. Method Validation Results

Calibration curves were established using multi-element standard solutions for each analyte. All ten elements displayed excellent linearity, with correlation coefficients (*r*) equal to or exceeding 0.9990. The limits of detection (LOD) and quantification (LOQ) for ICP-MS ranged from 0.0002 to 0.0016 μg·L^−1^ and from 0.0005 to 0.0052 μg·L^−1^, respectively; for ICP-AES, the corresponding ranges were 0.0341 to 0.0747 μg·L^−1^ and 0.1136 to 0.2490 μg·L^−1^. Instrument precision, represented as the relative standard deviation (RSD) of six consecutive injections of a standard solution, was below 2.0% for all elements. Method repeatability, evaluated by analyzing six independently prepared sample solutions from the same batch, yielded RSD values of less than 6.0%. Spike recovery tests conducted at three concentration levels provided mean recoveries ranging from 95.63% to 104.4%, with RSD values below 8.0% (Table 1). These results indicate that the method is sufficiently accurate and precise for the determination of inorganic element contents in LJ and LFS samples.

### 2.2. Determination of Inorganic Element Contents

The concentrations of ten inorganic elements (Fe, Cu, Mn, Zn, Na, Cr, Mo, Ni, As, and Cd) in LJ and LFS samples from various provenance were analyzed using ICP-MS and ICP-AES techniques. The results are detailed in Tables S3 and S4 included in the Supplementary Materials.

### 2.3. Hierarchical Clustering and Intraspecific Provenance Comparisons

**Figure 1:**
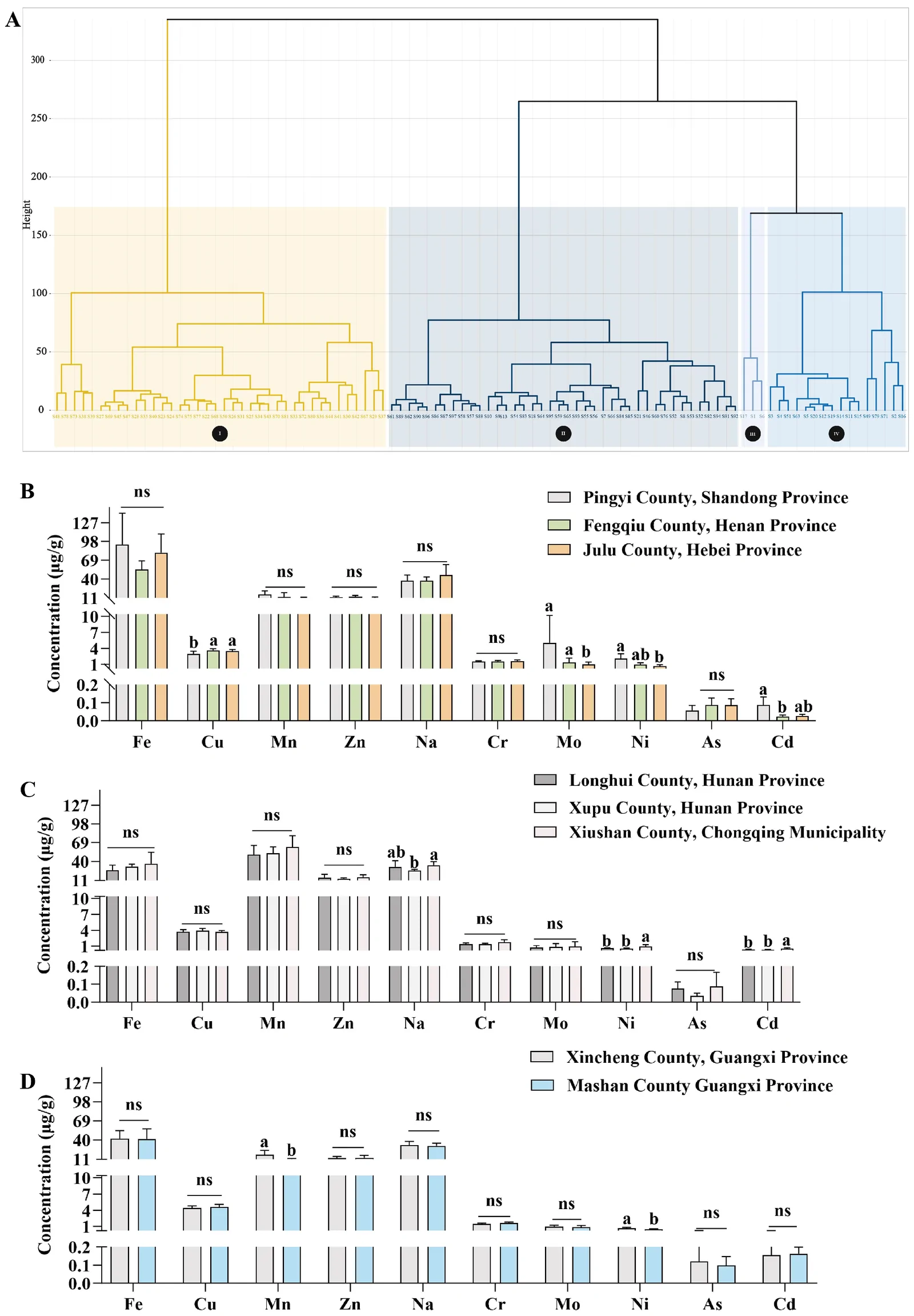
Hierarchical cluster analysis and intra-specific elemental comparisons across provenances. (**A**) Ward’s clustering dendrogram (squared Euclidean distance, Z-score standardized) for 97 samples; (**B**) LJ (Pingyi, Shandong; Fengqiu, Henan; Julu, Hebei); (**C**) LM (Longhui, Hunan; Xupu, Hunan; Xiushan, Chongqing); (**D**) LH (Xincheng, Guangxi; Mashan, Guangxi). Different lowercase letters indicate significant differences among groups (*p* < 0.05); ns, indicates no significant difference (*p* ≥ 0.05).

Hierarchical clustering analysis was conducted to assess the distribution patterns of elemental profiles among the samples. The resulting dendrogram classified all samples into four major clusters (Figure 1A). Cluster I primarily comprised LM and LC samples. Cluster II was mainly composed of LH and LF samples, along with several LJ samples. Cluster III included only three LJ samples. Cluster IV was predominantly made up of LJ samples, although it also contained a small number of LM, LC, and LH samples. At the intraspecific level, samples from different provenances did not form distinct geographical subclusters. LM samples from Hunan and Chongqing were found within the same cluster, while LJ samples from Shandong, Henan, and Hebei were dispersed across multiple clusters. Likewise, LC samples collected from six counties in Guangdong did not display noticeable geographical clustering. LF samples from Anlong County, Guizhou, and LH samples from Xincheng and Mashan counties in Guangxi formed relatively compact groups within their respective clusters; however, the LH samples from these two counties were not separated by provenance.

Intraspecific comparisons were further conducted for LJ, LM, and LH, which included samples from two or more provenances. Significant differences were observed in Cu, Mo, Ni, and Cd among the LJ provenances (Figure 1B). For LM, Na, Ni, and Cd exhibited significant variation between provenances (Figure 1C). In LH, only Mn and Ni showed significant differences between the populations from Xincheng and Mashan (Figure 1D).

### 2.4. Intraspecific Mantel Tests and Species-Specific Partial Correlation Analyses

**Figure 2:**
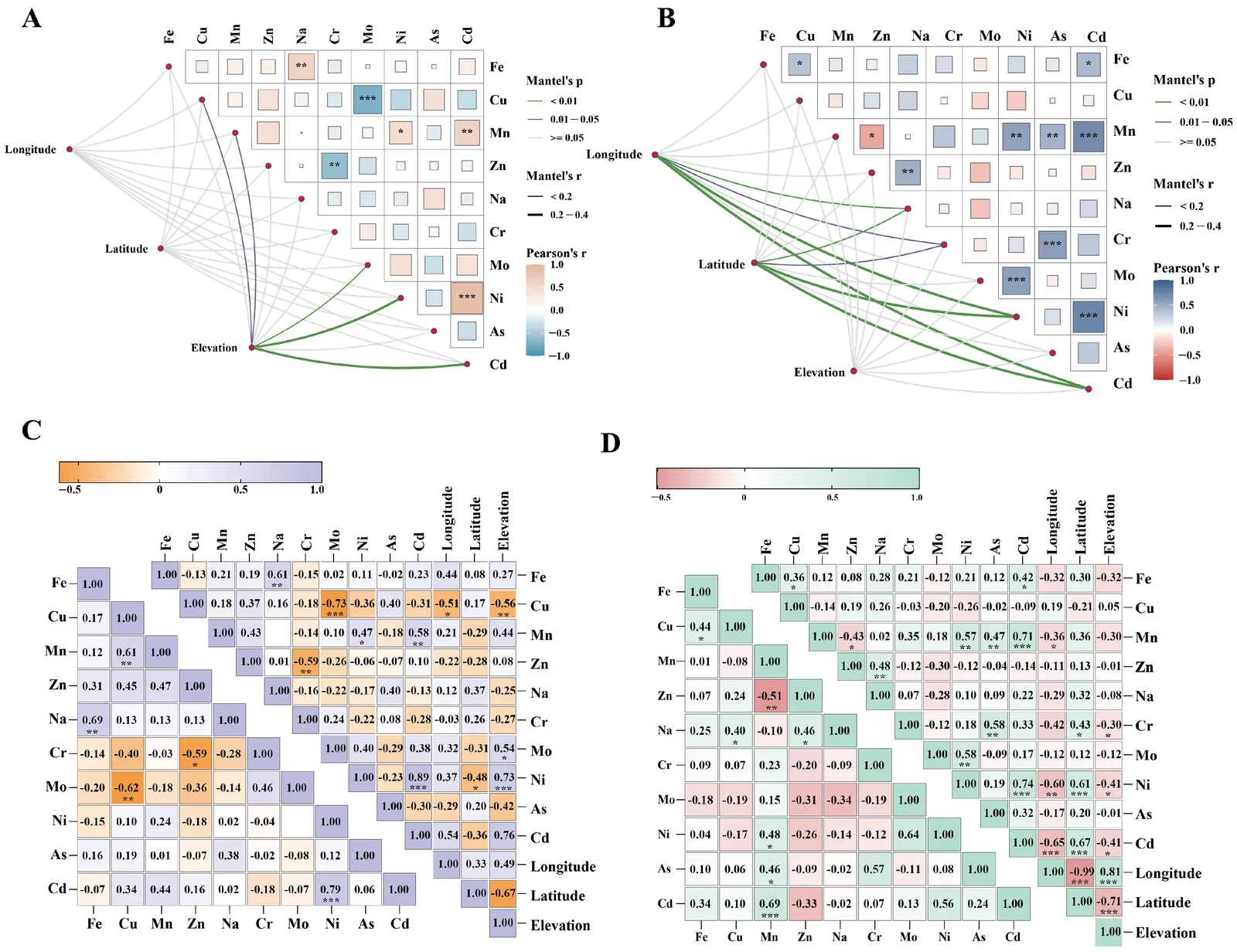
Species-specific mantel tests and partial correlation analyses. (**A**,**B**) mantel test network diagrams for (**A**) LJ and (**B**) LM; (**C**,**D**) partial correlation heatmaps (controlling for longitude, latitude, and elevation) for (**C**) LJ and (**D**) LM. The lower triangle displays partial correlation coefficients, while the upper triangle presents zero-order Pearson correlations. * *p* < 0.05, ** *p* < 0.01, *** *p* < 0.001.

To evaluate the independent influences of latitude, longitude, and elevation on elemental composition within specific species, intraspecific mantel tests were conducted. The species LJ was selected for this analysis due to its representation of three distinct provenances: Shandong, Henan, and Hebei, which create a clear elevational gradient. The species LM was also included, as it consists of two geographically separated populations from Hunan and Chongqing. The species LH, LC, and LF were excluded from subsequent analyses due to limited provenance representation or insufficient geographic variation. For the species LJ, the mantel test results indicated that elevation was significantly and positively correlated with Mo, Ni, and Cd (*p* < 0.01). Weaker but significant correlations were also observed between elevation and both Cu and Mn (*p* < 0.05). In contrast, neither longitude nor latitude demonstrated significant correlations with any element in LJ (Figure 2A). In the LM, significant positive correlations were found between longitude/latitude and Ni, Na, and Cd (*p* < 0.01), along with weaker but significant correlations with Cr (*p* < 0.05). Elevation did not exhibit significant correlation with any element in LM (Figure 2B).

To determine whether elemental correlations persist after controlling for the confounding effects of latitude, longitude, and elevation, species-specific partial correlation analyses were performed for LJ and LM, with all three geographical variables controlled concurrently. For LJ (Figure 2C), significant positive correlations remained for Fe-Na (*p* < 0.01), Ni-Cd (*p* < 0.001), and Cu-Mn (*p* < 0.01) after controlling for geographical variables, while Cu-Mo (*p* < 0.01) and Zn-Cr (*p* < 0.05) exhibited significant negative correlations. The correlations between Mn-Cd and Mn-Ni were no longer significant in the partial correlation analysis. Additionally, the Cu-Mn correlation, which was not significant in the original Pearson correlation, became significant after controlling for geographical variables. In the case of LM (Figure 2D), significant positive correlations persisted for Fe-Cu (*p* < 0.05), Cu-Na (*p* < 0.05), Mn-Cd (*p* < 0.001), Mn-As (*p* < 0.05), Mn-Ni (*p* < 0.05), Zn-Na (*p* < 0.05), Ni-Cd (*p* < 0.01), Cr-As (*p* < 0.01), and Mo-Ni (*p* < 0.001) after controlling for geographical variables. Moreover, Mn and Zn demonstrated a significant negative correlation (*p* < 0.01). Fe showed a significant partial correlation only with Cu, whereas Mn exhibited correlations with Cd, Ni, As, and Zn. The partial correlation between Mn and Zn (*r* = −0.506, *p* < 0.01) was stronger than their original Pearson correlation (*r* = −0.427, *p* < 0.05). The Fe-Cd correlation was no longer significant after controlling for geographical variables, while Cu–Na correlation became significant only in the partial correlation analysis (*r* = 0.397, *p* < 0.05).

### 2.5. Identification and Screening of Characteristic Differential Elements

**Figure 3:**
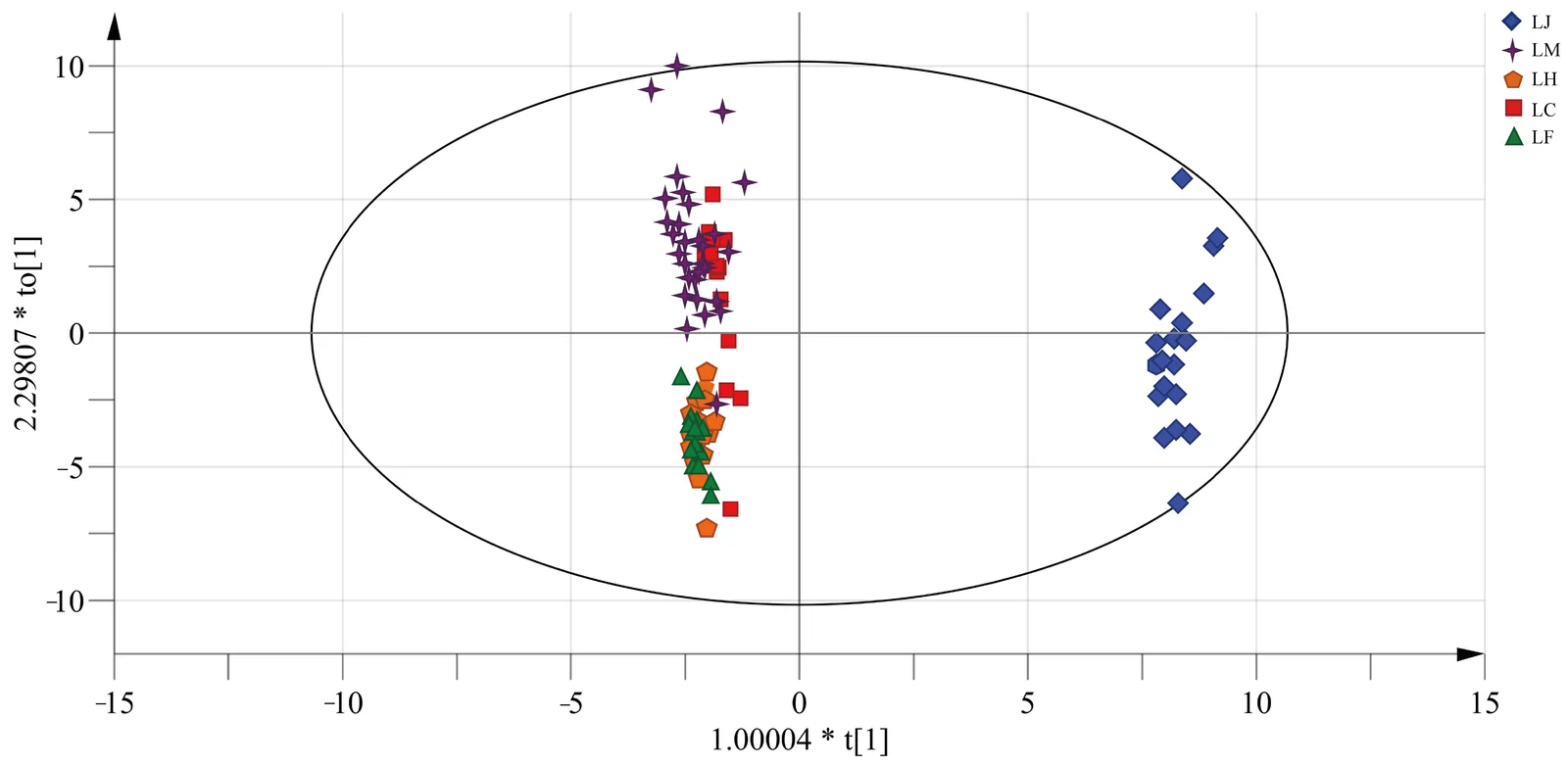
OPLS-DA score plot for LJ and LFS based on the concentrations of ten inorganic elements. Model parameters: *R*^2^*X* = 0.986, *R*^2^*Y* = 0.992, *Q*^2^ = 0.990. The ellipse represents the Hotelling’s T^2^ 95% confidence interval. The asterisks (*) in the axis label are part of the software-generated score component notation.

OPLS-DA was conducted to distinguish between LJ and LFS based on the concentrations of ten inorganic elements. The score plot (Figure 3) indicated that LJ samples were primarily positioned in the positive region of the *X*-axis and were distributed across both the positive and negative regions of the *Y*-axis. In contrast, LFS samples were predominantly located in the negative region of the *X*-axis, also spanning both sides of the *Y*-axis. Within the LFS group, LM and LC samples clustered in the positive region of the *Y*-axis, with a few LC outliers visible in the negative region; whereas LF and LH samples were mainly concentrated in the negative *Y*-axis region. The OPLS-DA model effectively separated the LJ and LFS groups.

**Figure 4:**
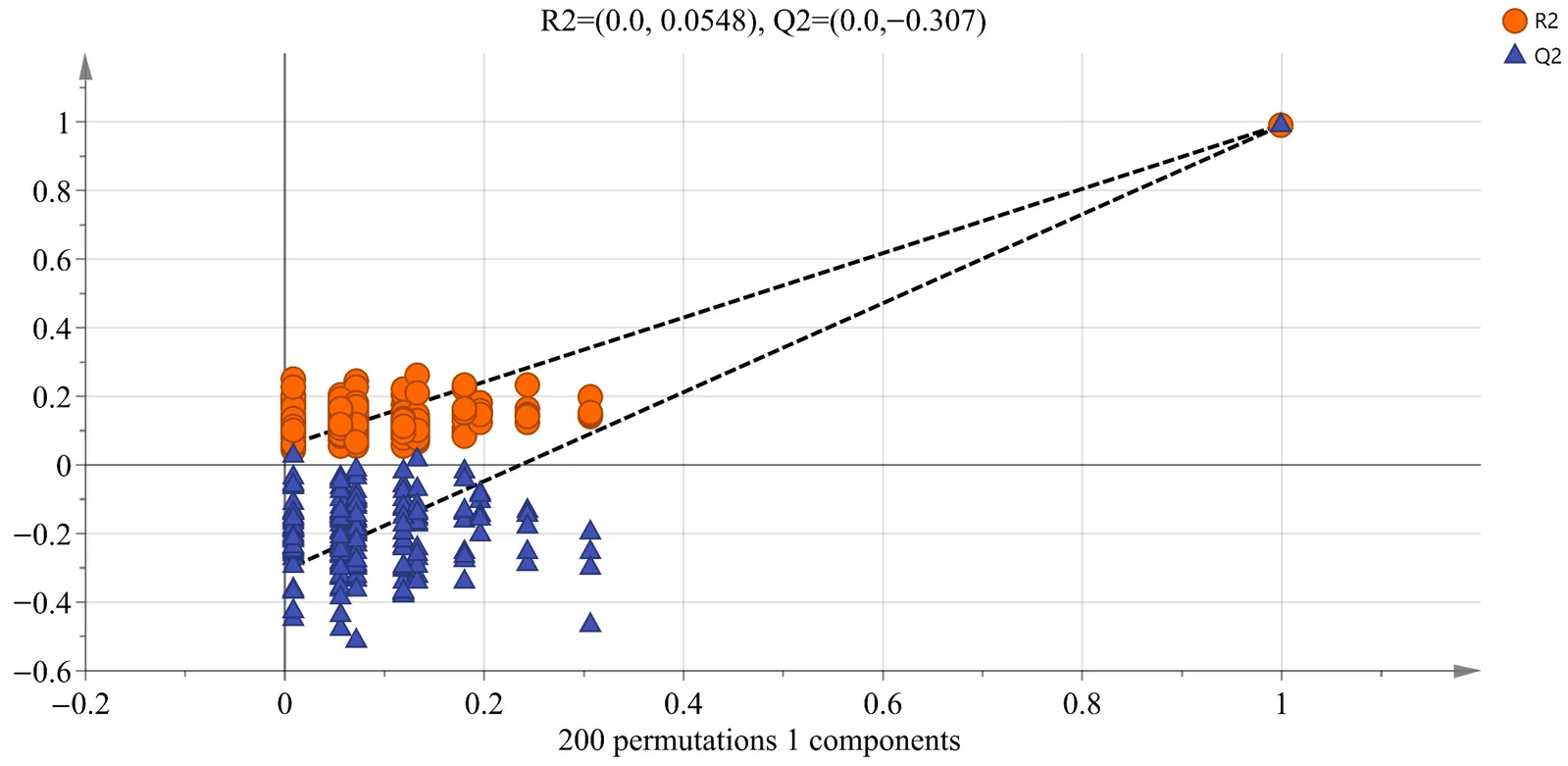
Validation of the OPLS-DA model through 200-permutation testing to differentiate between LJ and LFS based on ten inorganic elements. The X-axis represents the correlation between the original Y-variable and the permuted Y-variable; the Y-axis display the values of *R*^2^*Y* (goodness-of-fit) and *Q*^2^ (predictive ability). The dashed lines represent the regression lines for *R*^2^*Y* (orange) and *Q*^2^ (blue).

The OPLS-DA model demonstrated robust performance, with *R*^2^*X* = 0.986, *R*^2^*Y* = 0.992, and *Q*^2^ = 0.99, all exceeding the acceptable threshold of 0.5. Model validity was further confirmed by a 200-permutation test: the *Q*^2^-intercept was negative, indicating no overfitting (Figure 4). The model was developed using the present dataset and has not been externally validated on an independent sample set.

**Figure 5:**
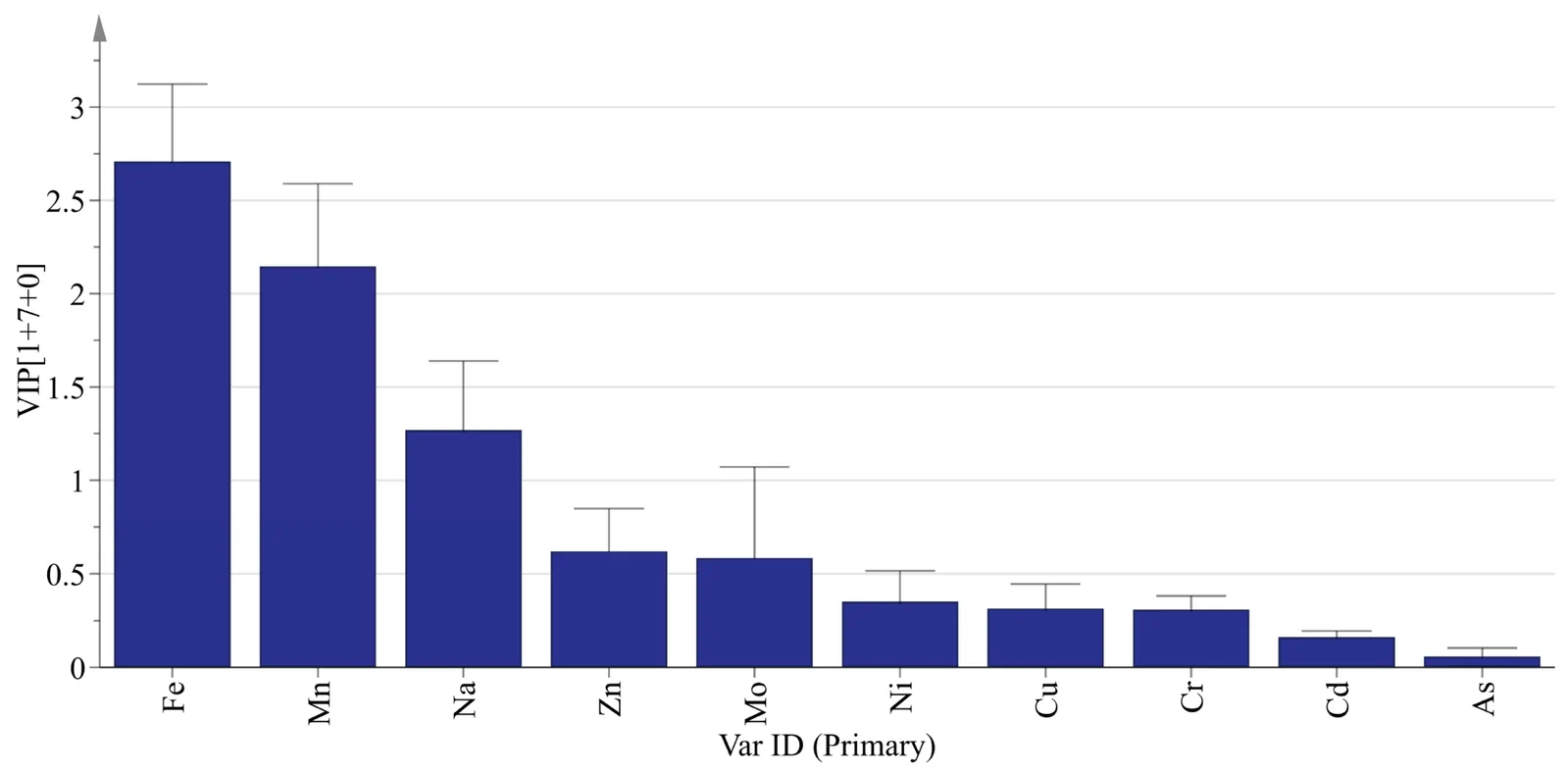
OPLS-DA variable importance in projection (VIP) plot for ten inorganic elements discriminating LJ from LFS. The VIP score reflects the contribution of each element to the separation between groups.

Using a VIP threshold of ≥1.0, three characteristic elements—Fe, Mn, and Na—were identified from the VIP score ranking plot (Figure 5) in descending order of contribution. Among these elements, Fe and Mn exhibited significantly greater contributions to the discrimination compared to Na, indicating that they are the most reliable markers for differentiating LJ from LFS, while Na plays a minor role.

### 2.6. Comparison of the Characteristic Differential Elements

**Figure 6:**
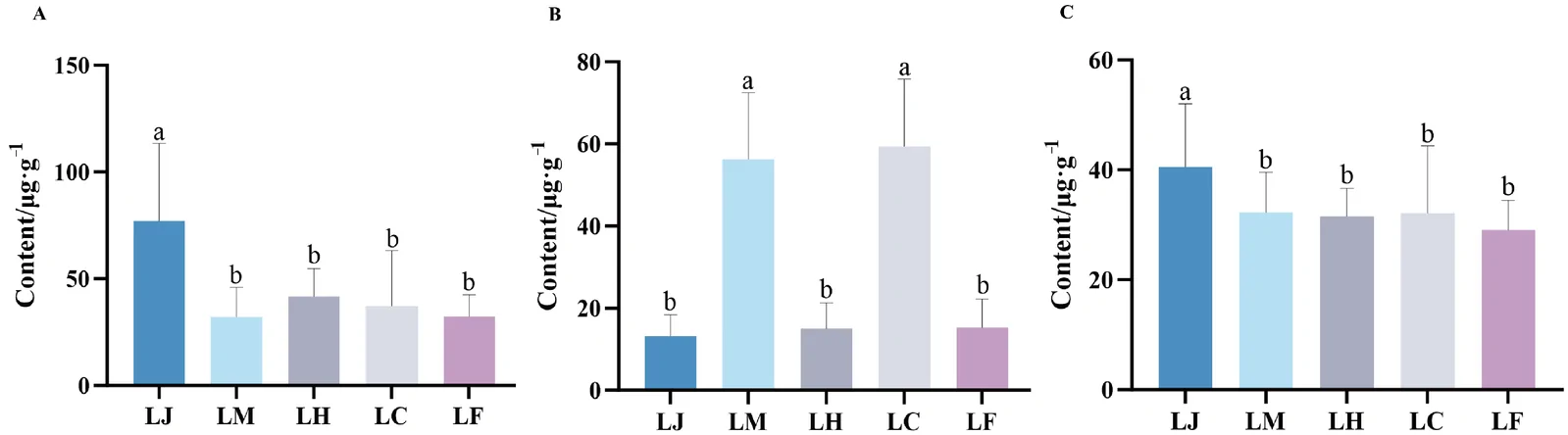
Comparison of key differential elemental contents among five Lonicera taxa. (**A**) Iron (Fe), (**B**) Manganese (Mn), and (**C**) Sodium (Na). Data are presented as mean ± standard deviation (SD) of biological replicates (LJ, *n* = 20; LM, *n* = 30; LH, *n* = 16; LC, *n* = 15; LF, *n* = 16). Statistical significance was evaluated using the Kruskal–Wallis H test followed by Dunn’s post hoc test with Bonferroni correction for multiple comparisons. Different lowercase letters indicate significant differences among groups (*p* < 0.05).

The quantitative results for Fe, Mn and Na in LJ and samples from various botanical origins of LFS are presented in Figure 6A–C. The Fe levels in LJ were significantly higher than those found in all LFS sources, whereas Mn presented a contrasting pattern, with notably higher concentrations in LM and LC compared to LJ. Likewise, Na levels in LJ were significantly elevated compared to the four LFS samples. Collectively, these findings establish distinct inorganic elemental profiles: LJ is characterized by high Fe, high Na, and low Mn, while LFS demonstrates the opposite trend, characterized by low Fe, low Na, and high Mn, especially in LM and LC.

### 2.7. Fe/Mn Ratio and Cluster Analysis

**Figure 7:**
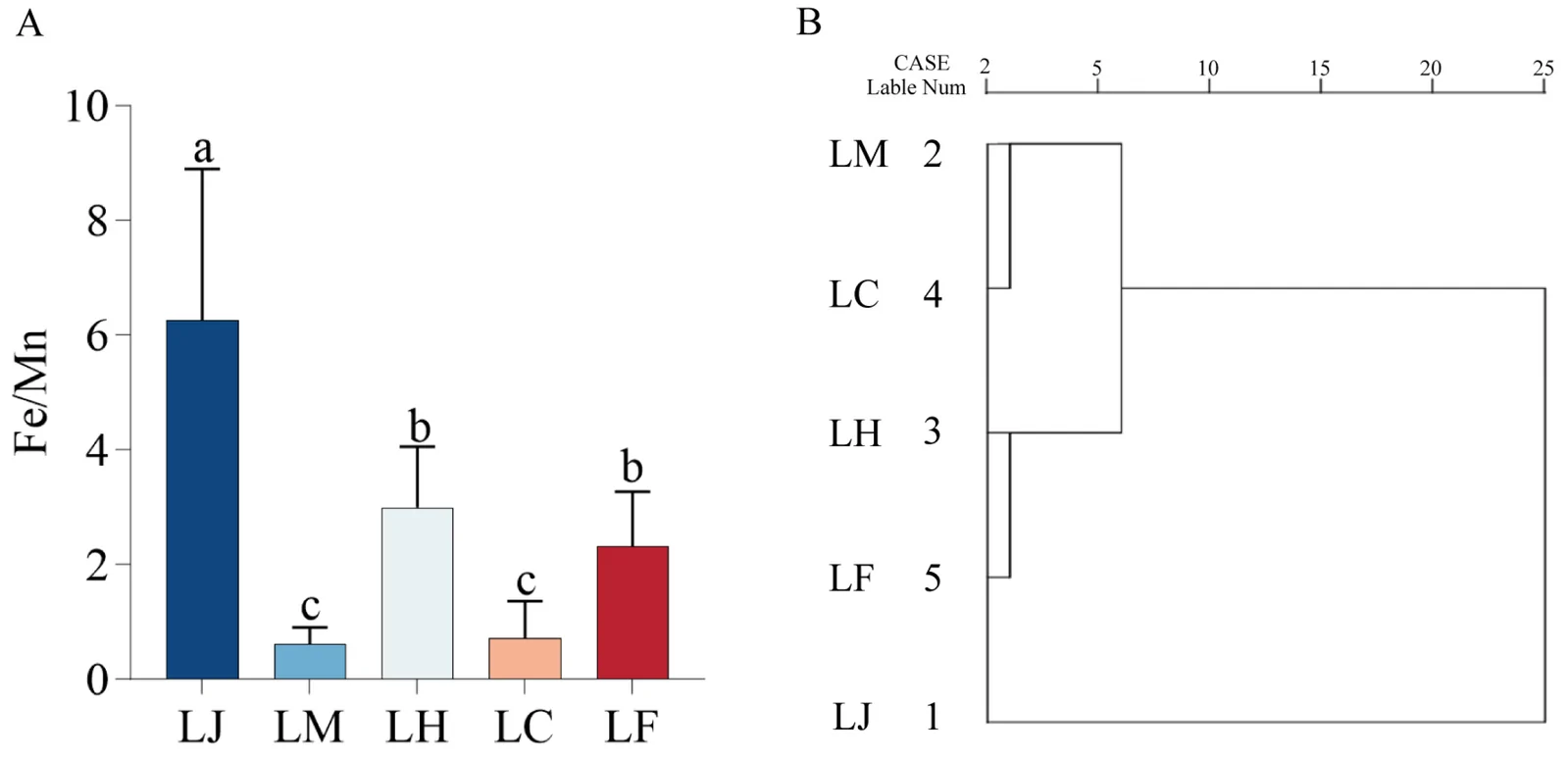
Analysis of Fe/Mn Ratio. (**A**) Comparison of Fe/Mn ratios among LJ and the four LFS taxa. (**B**) Hierarchical cluster analysis (Ward’s method) based on the Fe/Mn ratios. Different lowercase letters indicate significant differences among groups (*p* < 0.05).

As shown in Figure 7A, the Fe/Mn ratio was significantly higher in LJ compared to the four LFS groups. Notable inter-origin differences were also observed among the LFS samples. Specifically, the ratios in LH and LF were significantly higher than those in LM and LC, whereas no significant differences were found between LH and LF, or between LM and LC.

### 2.8. Network Pharmacological Analysis

#### 2.8.1. Candidate Targets for Inorganic Elements, Fe and Mn

**Figure 8:**
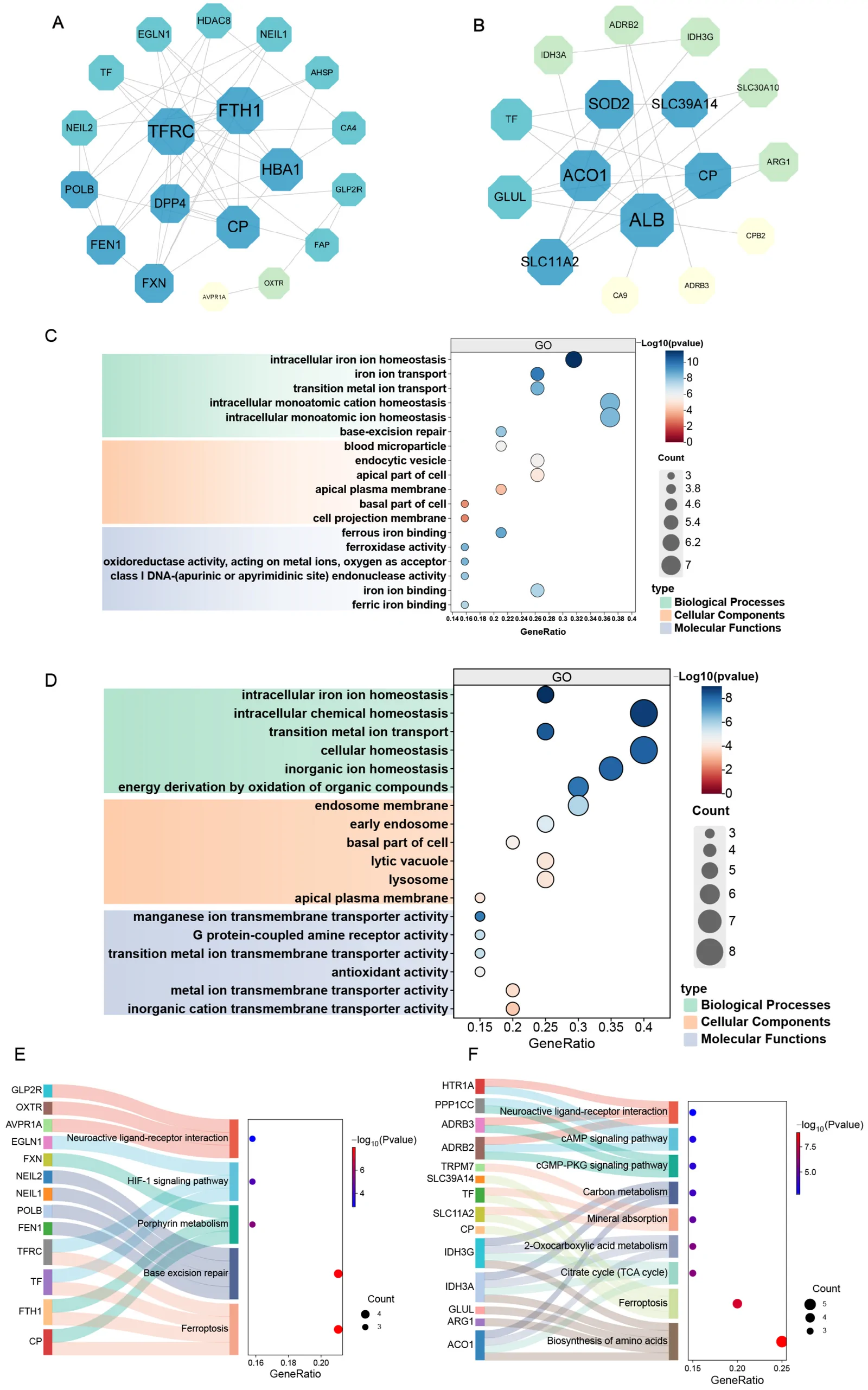
Network pharmacology analysis of Fe and Mn. (**A**) PPI network diagram for Fe and (**B**) Mn enrichment targets, nodes are colored according to degree values, with darker colors indicating higher degree; (**C**) results of GO enrichment analysis for Fe and (**D**) Mn; (**E**) results of KEGG enrichment analysis for Fe and (**F**) Mn.

A total of 20 targets were identified for iron (Fe) and 21 for manganese (Mn). The protein–protein interaction (PPI) networks for Fe- and Mn-related targets were constructed using Cytoscape 3.10.0 (Figure 8A,B). Based on the ranking of degree values, the top five targets for Fe were ferritin heavy chain 1 (FTH1), transferrin receptor 1 (TFRC), hemoglobin subunit alpha 1 (HBA1), ceruloplasmin (CP), and dipeptidyl peptidase 4 (DPP4). For Mn, the top five targets included serum albumin (ALB), aconitase 1 (ACO1), superoxide dismutase 2 (SOD2), CP, and solute carrier family 39 member 14 (SLC39A14).

#### 2.8.2. Analysis of GO and KEGG Enrichment Results

**Figure 9:**
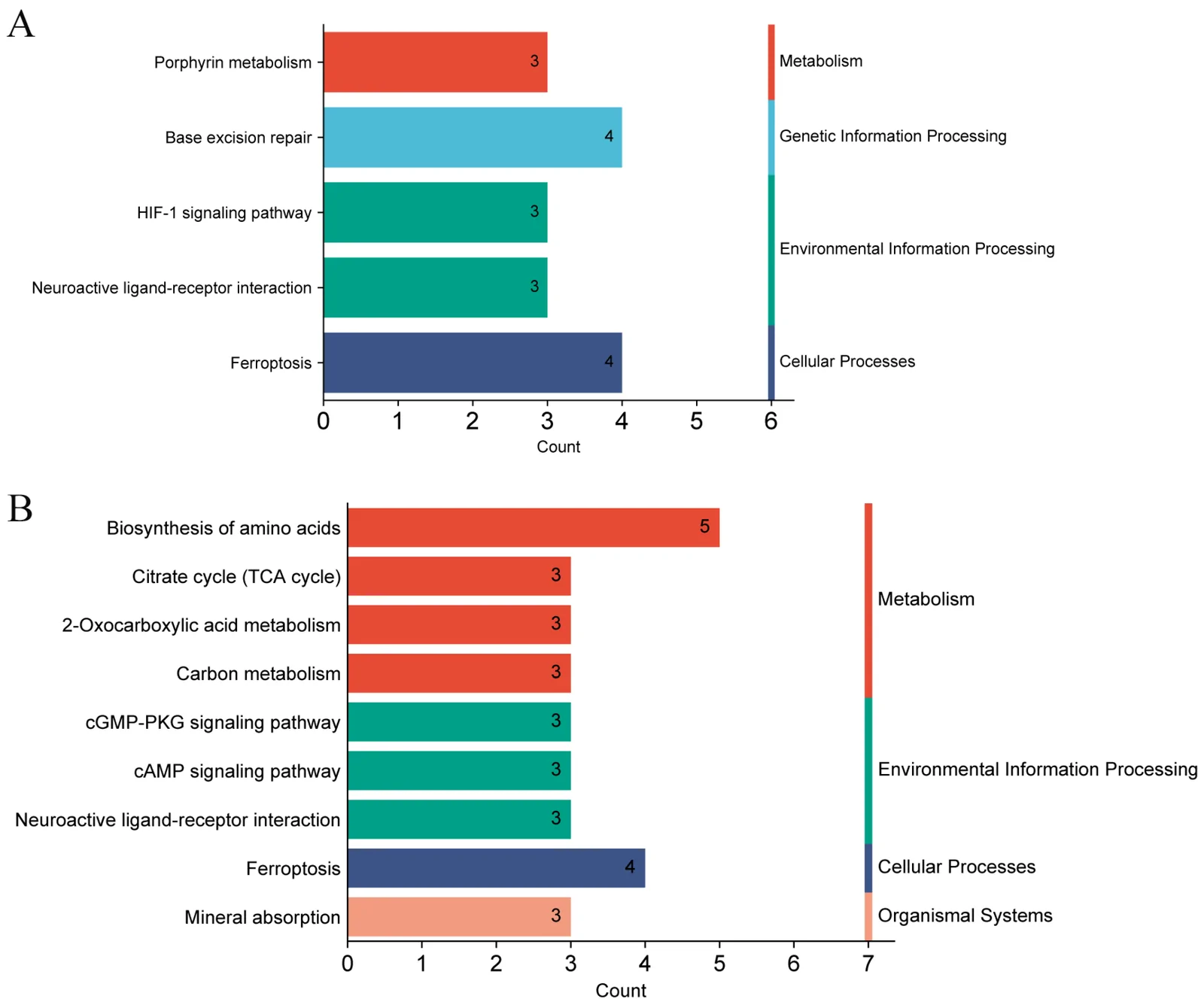
KEGG pathway secondary classification for iron-associated targets (**A**) and manganese-associated targets (**B**). The x-axis represents the number of enriched target genes in each functional category, while the y-axis lists the KEGG functional categories. Different colors denote distinct KEGG pathway classes. The length of each bar corresponds to the count of genes enriched in each category.

The results of the GO enrichment analysis for Fe are presented in Figure 8C, where a total of 68 GO entries were identified, comprising 39 biological processes (BP), 6 cellular components (CC), and 23 molecular functions (MF). Similarly, the GO enrichment analysis for manganese (Mn) is illustrated in Figure 8D, revealing 103 GO entries, which include 69 BP, 21 CC, and 13 MF. The results of the KEGG analysis indicated five pathways associated with Fe: ferroptosis, base excision repair, porphyrin metabolism, the HIF-1 signaling pathway, and neuroactive ligand-receptor interaction (Figure 8E). In contrast, the analysis for Mn identified 21 targets that corresponded to nine KEGG pathways, as shown in Figure 8F: biosynthesis of amino acids, ferroptosis, the citrate cycle (TCA cycle), 2-oxocarboxylic acid metabolism, mineral absorption, carbon metabolism, the cGMP-PKG signaling pathway, the cAMP signaling pathway, and neuroactive ligand-receptor interaction. Further categorization of the pathways is depicted in Figure 9A,B, where Fe was classified into the following categories: cellular processes (one entry), environmental information processing (two entries), genetic information processing (one entry), and metabolism (one entry). For Mn, the classifications included biological systems (one entry), cellular processes (one entry), environmental information processing (three entries), and metabolism (four entries).

### 2.9. Molecular Docking Results

**Figure 10:**
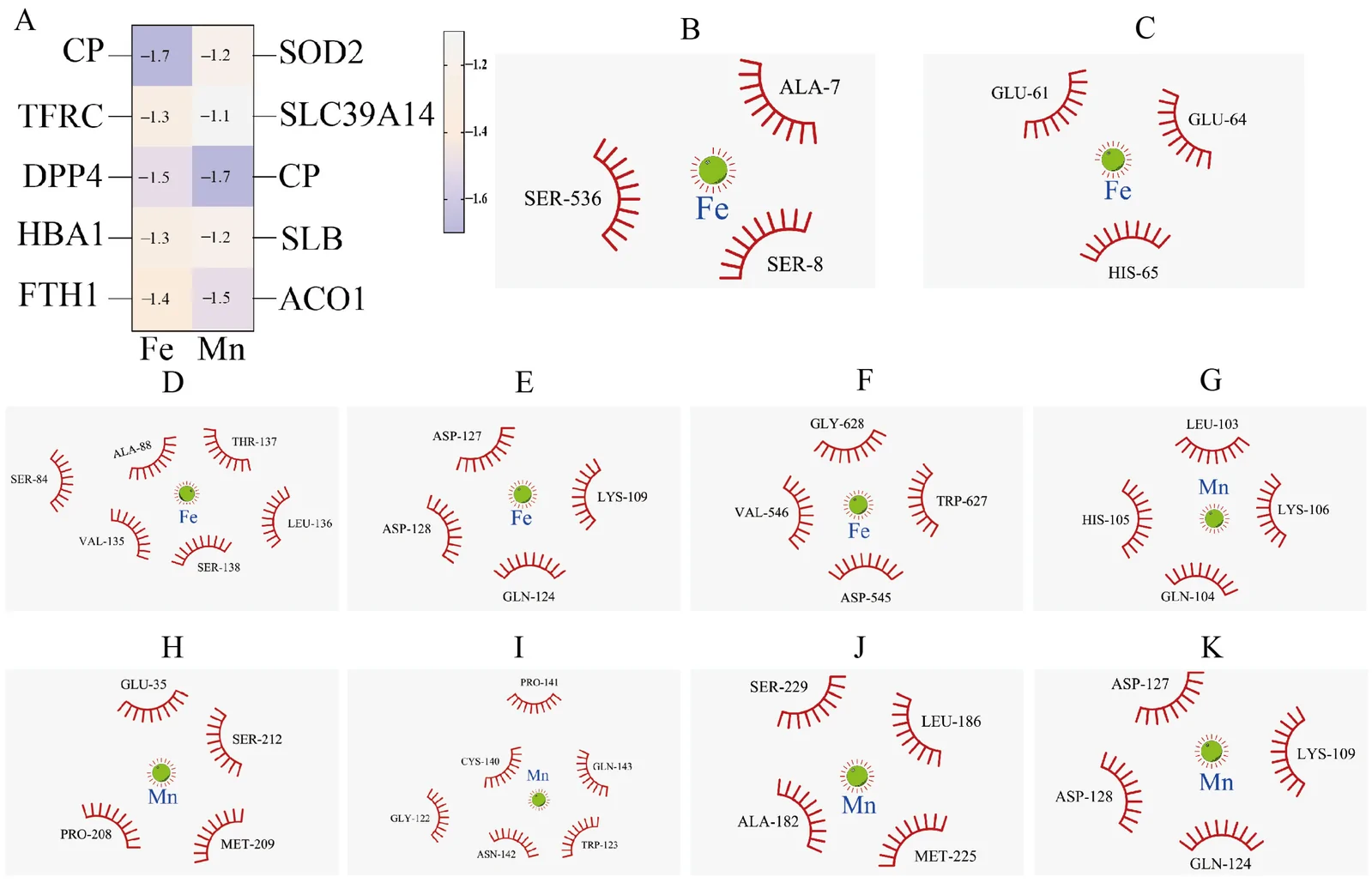
(**A**) Heatmap of simulated docking scores for Fe and Mn; more negative values indicate a better theoretical electrostatic match under static vacuum simulation conditions. (**B**–**F**) 2D schematic diagrams illustrate the distribution of pocket residues surrounding the Fe ion for TFRC, FTH1, HBA1, CP, and DPP4. (**G**–**K**) 2D schematic diagrams depict the distribution of pocket residues surrounding the Mn ion for ALB, ACO1, SOD2, SLC39A14, and CP. Note: All 2D diagrams display only the spatial positions of surrounding amino acids, without simulated coordination bonds.

Molecular docking was conducted separately for Fe and Mn against their respective five core targets. The targets for Fe included ferritin heavy chain 1 (FTH1), transferrin receptor (TFRC), hemoglobin subunit alpha 1 (HBA1), ceruloplasmin (CP), and dipeptidyl peptidase 4 (DPP4). The targets for Mn comprised albumin (ALB), aconitase 1 (ACO1), superoxide dismutase 2 (SOD2), CP, and solute carrier family 39 member 14 (SLC39A14), resulting in ten sets of receptor-ligand docking outcomes. The docking results summarized in Table 2 indicate that all predicted binding scores were negative under static vacuum simulation conditions. This finding was based on the positioning of metal ions within the protein pockets during the calculations (Figure 10A). Among all the tested targets, CP exhibited relatively lower predicted docking scores for both ligands, with a value of −1.7 kcal/mol, involving surrounding polar residues such as aspartic acid (ASP)-127, ASP-128, lysine (LYS)-109, and glutamine (GLN)-124. Fe yielded predicted docking scores with DPP4 (−1.5 kcal/mol) and FTH1 (−1.4 kcal/mol), primarily mediated by residues valine (VAL)-546 and glutamic acid (GLU)-61. In addition to CP, Mn yielded predicted docking scores with ACO1 (−1.5 kcal/mol), ALB (−1.2 kcal/mol), and SOD2 (−1.2 kcal/mol), facilitated by key residues such as serine (SER)-212, LYS-106, and asparagine (ASN)-142. Representative two-dimensional (2D) diagrams illustrating the distribution of residues surrounding Fe and Mn ions in the docking outputs are presented in Figure 10B–K.

## 3. Discussion

In this study, we conducted a quantitative analysis of ten inorganic elements present in LJ and LFS using ICP-MS and ICP-AES techniques. The results revealed significant differences in inorganic element content between the two species, aligning with previous findings [19,20]. OPLS-DA identified Fe, Mn, and Na as discriminatory elements, while cluster analysis based on the Fe/Mn ratio further distinguished the two groups. These preliminary findings suggest that the Fe/Mn ratio should be investigated as a potential discriminative indicator for LJ and LFS.

The accumulation of elements in plants is jointly regulated by genetic factors and environmental conditions. Species identity and geographical provenance are two important factors that may operate independently or be interrelated. Variations in soil type, altitude, climatic conditions, and element availability across regions can significantly influence the uptake and accumulation of inorganic elements in plants. The samples in this study encompassed a broad range of ecological regions and soil types, including brown soils in Shandong, sandy loams in Henan and Hebei, selenium-rich sandy loams and red soils in Hunan, yellow soils in Chongqing, calcareous and calcareous loam soils in Guangxi, red and purple soils in Guangdong, and yellow soils in Guizhou. This extensive coverage established a substantial geographical and ecological gradient. Consequently, the observed differences in elemental content may partially reflect the combined effects of soil element availability and plant adaptability under varying habitat conditions.

Intraspecific provenance comparisons within LJ and LM revealed that, despite significant environmental differences among populations (including altitude, soil type, and location), critical discriminating elements such as Fe and Mn remained relatively stable within each species. No significant inter-provenance differences in Fe or Mn were detected among LJ populations or between the Hunan and Chongqing populations of LM. This finding suggests a species-specific regulation of uptake, transport, and homeostasis of these elements. Conversely, certain trace elements displayed provenance-related patterns: Cu, Mo, Ni, and Cd in LJ; Na, Ni, and Cd in LM; and Mn and Ni in LH exhibited significant regional differences. This implies that environmental factors predominantly influence elements that are sensitive to soil conditions.

Mantel tests and partial correlation analyses further demonstrated that, after controlling for species identity and geographical variables, Fe and Mn exhibited relatively low sensitivity to geographical variation. However, their elemental association networks differed between species, indicating species-specific regulatory strategies. The long-term adaptation of the same species to varying ecological environments may contribute to the development of chemoecotypes, potentially through rhizosphere modulation and differential expression of element transporters [21].

The distinct accumulation patterns of these elements may reflect differences in the uptake and translocation of mineral nutrients between the two herbs. Fe and Mn are essential elements that participate in various enzymatic reactions and metabolic regulation. Variations in the concentrations of these elements may indirectly influence the accumulation of other active constituents and the pharmacological effects of medicinal materials [22,23]. Previous studies have suggested a potential association between Fe and Mn contents and the intrinsic properties of TCM, indicating that a higher Fe/Mn ratio correlates with a specific property classification, whereas a lower ratio aligns with an opposite classification [14,24]. Our elemental analysis revealed significant differences in the concentrations of Fe and Mn between LJ and LFS, which were identified by OPLS-DA as the primary factors driving inter-group separation. Although the model parameters (*R*^2^*X* = 0.986, *R*^2^*Y* = 0.992, *Q*^2^ = 0.990) demonstrate a strong discriminatory capacity, it is important to acknowledge that they are derived from internal cross-validation without incorporating an independent test set, thus leaving open the possibility of overfitting. The Fe/Mn ratio was also calculated from the same dataset, but without an established diagnostic cut-off. Consequently, it should be considered a candidate indicator that requires validation in independent cohorts before it can be applied as a reliable differentiating parameter for LJ and LFS. These differences may arise from genetic and environmental factors that influence mineral uptake and homeostasis, potentially affecting the accumulation of secondary metabolites by modulating key enzymatic pathways [25,26,27]. Incorporating inorganic elemental fingerprinting into the quality control framework could provide an additional dimension for species authentication; however, the practical utility of the Fe/Mn ratio as a standalone marker requires further investigation. Future studies should focus on elucidating the causal relationships among elemental composition, metabolic regulation, and therapeutic efficacy, considering the Fe/Mn ratio as one of several potential discriminative indicators that merit further exploration.

Network pharmacology and molecular docking were utilized as exploratory bioinformatics tools to preliminarily investigate potential biological correlations related to differential levels of Fe and Mn. Pathway enrichment analysis indicated that Fe-associated candidate targets were enriched in the pathways of ferroptosis and porphyrin metabolism, while Mn-related hubs predominantly clustered around energy metabolism pathways, including the TCA cycle. These enrichment profiles align with existing literature reports on trace element homeostasis modules [16,17], providing preliminary network-level insights into the potential biological differences induced by varying levels of Fe and Mn between the two herbs. It is important to note that this analysis relies on public database annotations of metal-binding proteins, which are primarily designed for organic ligands. Therefore, the screened targets should be regarded as candidate associations rather than confirmed in vivo interaction pairs.

Molecular docking simulations produced negative binding energy values under static vacuum conditions, indicating favorable electrostatic interactions between Fe/Mn ions and the target pocket residues. AutoDock Vina’s native force field is optimized for organic small molecules and metal chelate complexes, which lacks dedicated parameters for accurately simulating metal coordination bonds and aqueous hydration environments. Consequently, the calculated affinities and pocket poses represent simplified in silico predictions rather than definitive physiological binding modes. The 2D pocket diagrams clearly illustrate the distribution of residues surrounding the metal ions, providing auxiliary reference for the spatial matching relationship between these elements and core proteins. Collectively, the integrated bioinformatics results offer a preliminary hypothetical framework for subsequent functional verification; however, relevant correlations related to oxidative stress and metabolic regulation require supporting cellular or animal experiments to draw definitive conclusions. Based on these findings, we tentatively propose that varying Fe and Mn homeostasis may be associated with the distinct biological characteristics of the two herbs, and follow-up functional studies could focus on this perspective.

It is important to note that according to the Chinese Pharmacopoeia (2025 Edition) [1], LJ is subject to statutory limits for heavy metals (Cd ≤ 1 mg/kg; As ≤ 2 mg/kg), while no such limits are currently established for LFS. In the dataset analyzed, all LJ samples were found to be in compliance with these thresholds. However, certain specimens of LM exhibited significantly higher concentrations of Cd compared to those measured in LJ. Given the widespread use of *Lonicera* species as health teas and food additives, the lack of defined heavy metal standards for LFS represents a regulatory gap that requires systematic monitoring and future safety evaluations.

Several limitations of this study should be noted. Samples were rinsed with tap water followed by double-distilled water before digestion, a process that may leach readily soluble endogenous minerals and skew the quantified levels of sodium. The sampling distribution of LJ and LFS subspecies was geographically unbalanced, and matched soil physicochemical indicators, such as pH, organic matter, and available trace elements, were not collected alongside the herbal samples. This limitation restricts the accurate discrimination of genetic and soil-mediated effects on element accumulation. Additionally, we quantified only total elemental concentrations through complete acid digestion, failing to test water-soluble decoction fractions and gastrointestinal bioaccessible forms of Fe and Mn. Furthermore, all network pharmacology and molecular docking results are based solely on computational predictions, lacking validation through cellular or animal functional assays. To address these gaps, future research should expand the geographically balanced sample coverage of LJ and all four varieties of LFS, utilize paired soil–herb simultaneous detection to disentangle the genetic and environmental drivers of elemental variation, conduct aqueous decoction extraction and simulated gastrointestinal digestion tests to assess the actual bioavailability of characteristic trace elements, and implement in vitro cell functional experiments targeting oxidative stress and energy metabolism pathways to validate the tentative regulatory associations predicted by bioinformatics workflows.

## 4. Materials and Methods

### 4.1. Chemicals

Certified reference standards of the ten target elements (Fe, Cu, Mn, Zn, Na, Cr, Mo, Ni, As, and Cd) were purchased from the National Institute of Metrology (Beijing, China). Analytical-grade nitric acid (≥68%) and hydrogen peroxide (H_2_O_2_) (≥30%) were supplied by Beijing Chemical Works (Beijing, China). Ultra-pure water was prepared using a Milli-Q system (Millipore, Bedford, MA, USA).

### 4.2. Plant Material

A total of 97 dried bud samples were collected between May and June 2021: LJ (Shandong, Henan, and Hebei; *n* = 20), LM (Hunan and Chongqing; *n* = 30), LH (Guangxi; *n* = 16), LC (Guangdong; *n* = 15), and LF (Guizhou; *n* = 16). Detailed sampling information, including GPS coordinates, altitude, soil type, and collection date, is presented in Table S1 (Supplementary Materials). The sampling locations encompassed a wide range of ecological gradients (elevation: 5–1368 m), with predominant soil types including brown soil, sandy loam, selenium-rich sandy loam, red soil, yellow soil, limestone soil, calcareous soil, purple soil, and mountain yellow soil. All specimens were collected under clear, sunny conditions from cultivated or semi-cultivated plants (4–6 years old, adult stage) that were maintained according to standard local agricultural practices, without the use of specialized agronomic interventions or fertilization. After harvest, samples were naturally air-dried and stored in dry conditions until analysis, with all elemental determinations conducted within six months of collection. All plant materials were verified by Professor Weidong Li (School of Chinese Materia Medica at Beijing University of Chinese Medicine).

### 4.3. Sample Preparation and Microwave Digestion

Before digestion, the air-dried samples were rinsed with running tap water for approximately 2 min to remove surface debris, followed by three washes with double-distilled water (ca. 50 mL each). The samples were then dried at 60 °C until they reached a constant weight and ground into a fine powder using an agate mortar. An accurate weight of 200 mg of each sample was transferred to a polytetrafluoroethylene (Teflon) digestion vessel (Beijing Zhengcheng Biotechnology Co., Ltd., Beijing, China). Subsequently, 5 mL of 70% HNO_3_ was added, and the mixture was allowed to stand at room temperature overnight for predigestion. Following this, 2 mL of 30% H_2_O_2_ was added, and the mixture was left at room temperature for 20 min before the vessels were sealed. The digestion procedure was adapted from the methods described by Bora et al. [28] and Xie et al. [29] with minor modifications. The samples were digested in a microwave digestion system (MARS, CEM, Matthews, NC, USA) operated at a power of 1600 W. The temperature program was as follows: ramp to 120 °C at a rate of 9 °C/min and hold for 1 min; ramp to 160 °C at a rate of 8 °C/min and hold for 5 min; then ramp to 185 °C at a rate of 8 °C/min and hold for 25 min. Complete operating parameters for microwave digestion are provided in Table S2 (Supplementary Materials).

After cooling to room temperature, the digests were diluted to 50 mL with ultra-pure water in the digestion vessels. The solutions were then filtered through qualitative filter paper, and 10 mL aliquots were collected for elemental determination using ICP-AES or ICP-MS.

### 4.4. ICP-AES and ICP-MS Analysis

Elemental analysis was conducted using the Agilent 7000X ICP-MS (Agilent Technologies, Santa Clara, CA, USA) and the iCAP 6000 ICP-AES (Thermo Scientific, Waltham, MA, USA). The operating conditions for the ICP-MS were as follows: RF power of 1.15 kW, auxiliary gas flow rate of 0.5 L/min, sample uptake time of 30 s, and pump speed of 40 rpm. The operating conditions for the ICP-AES were: RF power of 1.15 kW, frequency of 27.12 MHz, carrier gas flow rate of 1.0 L/min, sample uptake rate of 1.85 mL/min, long-wavelength integration time of 5 s, and short-wavelength integration time of 30 s. The ICP-AES was utilized for elements with relatively high sample concentrations (Fe, Cu, Mn, Zn, Na), while the ICP-MS was used for trace-level elements (Cr, Mo, Ni, As, Cd) that required greater detection sensitivity.

### 4.5. Method Validation

#### 4.5.1. Linearity, Detection Limit, and Quantification Limit

A series of mixed standard solutions at various concentration levels was injected, and calibration curves were constructed by plotting the concentration of each element against its corresponding response value. The blank solution was analyzed 11 times; the limit of detection (LOD) was defined as the concentration corresponding to three times the standard deviation of the blank response, while the limit of quantification (LOQ) was defined as the concentration corresponding to ten times the standard deviation.

#### 4.5.2. Precision and Repeatability

Instrument precision was evaluated by conducting six consecutive injections of a mixed standard solution. Method repeatability was assessed by analyzing six independently prepared sample solutions derived from the same representative sample.

#### 4.5.3. Spike Recovery

Spike recovery experiments were conducted by adding known amounts of mixed standard solutions to the samples at three concentration levels (approximately 50%, 100%, and 150% of the native content). For each concentration level, three spiked samples were prepared and analyzed according to the same procedure described above.

### 4.6. Network Pharmacology Analysis

#### 4.6.1. Collection of Fe and Mn Action Targets

Network pharmacology has been utilized to characterize the multi-target regulation of essential trace elements such as Fe, Mn, Zn, and Cu [16,17]. In this study, we retrieved potential proteins associated with Fe and Mn from DrugBank (https://go.drugbank.com/, 28 November 2024) and the Therapeutic Target Database (TTD) (https://ttd.idrblab.cn/, 28 November 2024). Although these databases primarily record small-molecule therapeutics, they include annotations for metal transporters, metal-binding enzymes, and genes involved in ion homeostasis, which serve as a preliminary screening mechanism for inorganic ion targets. Given the unique interactions between metals and proteins, we complemented the database screening with manual literature curation to include only experimentally verified metal-binding proteins, such as SLC transporters, transferrin, and ferritin. The final target set integrates outputs from the databases with experimental evidence. Importantly, these targets were used to construct a hypothetical systemic regulatory network instead of verifying direct physical interactions between metal ions and proteins, and all candidates were utilized for subsequent analyses.

#### 4.6.2. Constructing Protein–Protein Interaction (PPI) Networks for Fe and Mn-Related Targets

The Fe- and Mn-related targets were submitted to the STRING database (https://string-db.org/, 28 November 2024) with the species limited to Homo sapiens. Fe, the initial search, yielded a limited number of targets at the default medium confidence threshold (≥0.4). Therefore, the threshold was lowered to 0.15 (low confidence) to include a sufficient number of interactors for network construction. In contrast, the default medium confidence threshold (≥0.4) was maintained for Mn. The resulting PPI network was subsequently filtered and visualized using Cytoscape version 3.10.0 (Cytoscape Consortium, San Diego, CA, USA), and topological parameters, such as degree, were calculated using the Network Analyzer plugin.

#### 4.6.3. Gene Ontology (GO) and Kyoto Encyclopedia of Genes and Genomes (KEGG) Enrichment Analyses

GO and KEGG pathway enrichment analyses of the identified targets were conducted using the Metascape database (https://metascape.org/, 28 November 2024). The enrichment results, including BP, CC, MF, and KEGG pathway annotations, were retrieved. The most significantly enriched GO terms and KEGG pathways were selected and visualized.

### 4.7. Molecular Docking

The three-dimensional (3D) structures of all target proteins, except for SLC39A14, were obtained from the RCSB Protein Data Bank (PDB; https://www.rcsb.org/, 23 June 2026). The 3D structure of SLC39A14 was acquired from the AlphaFold Protein Structure Database (UniProt ID: Q15043; computationally predicted model). The 2D structures of the ligands (Fe and Mn) were downloaded from the PubChem database (https://pubchem.ncbi.nlm.nih.gov/, 23 June 2026). Prior to docking, all protein structures were preprocessed using PyMOL 3.0 (Schrödinger, Inc., New York, NY, USA) to eliminate crystallographic water molecules and non-cognate endogenous ligands. The prepared protein and ligand structures were subsequently imported into AutoDock Tools 1.5.7 (Scripps Research, La Jolla, CA, USA) for additional preparation, which included the addition of polar hydrogens, assignment of Gasteiger partial charges, calculation of the geometric centers of the ligands, and definition of rotatable bonds. Grid boxes were configured to encompass the potential binding pockets of each target protein, with detailed center coordinates and box dimensions summarized in Table 2. Molecular docking was conducted using AutoDock Vina 1.2.4 (Scripps Research, La Jolla, CA, USA) to assess the theoretical binding affinities between the metal ions and the target proteins. It is crucial to note that AutoDock Vina utilizes a semi-empirical force field primarily optimized for organic ligands, which presents inherent limitations in accurately modeling the coordination chemistry of transition metal ions. As a result, the findings reported here should be interpreted strictly as preliminary in silico screenings of spatial and electrostatic complementarity, consistent with methodological precedents for metal-containing complexes documented in the literature [30]. To accommodate the ionic characteristics of the ligands, we manually adjusted the atomic charges and radii of Fe and Mn during the preparation phase in AutoDock Tools. The specific coordination geometries and binding mechanisms necessitate further investigation through advanced quantum mechanical/molecular mechanical (QM/MM) simulations or experimental structural biology techniques.

### 4.8. Statistical Analysis

Ninety-seven independent biological samples were included in the study: LJ (*n* = 20), LM (*n* = 30), LH (*n* = 16), LC (*n* = 15), and LF (*n* = 16). Each sample was digested and analyzed in triplicate technical replicates using ICP-MS/ICP-AES, with the mean value utilized for subsequent calculations. The data are presented as mean ± SD. Normality and homogeneity of variances were assessed using the Shapiro–Wilk and Levene’s tests, respectively. Cu, meeting both parametric assumptions, was compared among groups using one-way anova followed by Tukey’s honest significant difference (HSD) test. The remaining nine elements (Fe, Mn, Zn, Na, Cr, Mo, Ni, As, and Cd) violated normality; therefore, they were analyzed using the Kruskal–Wallis H test with Dunn’s post hoc test and Bonferroni correction applied. Hierarchical cluster analysis was conducted using ward’s method with squared Euclidean distance on z-score standardized data. Species-specific mantel tests were performed on LJ and LM using Bray–Curtis distances calculated from the ten element concentrations and Euclidean distances calculated from standardized longitude, latitude, and elevation; significance was evaluated through 999 permutations. Partial correlation analyses were executed on LJ and LM by calculating partial correlation coefficients among the ten elements while simultaneously controlling for longitude, latitude, and elevation. OPLS-DA was carried out on pareto-scaled data using SIMCA version 14.1 (Sartorius Stedim Data Analytics AB, Umeå, Sweden), with model performance assessed through *R*^2^*X*, *R*^2^*Y*, and *Q*^2^, and validated via 200-permutation tests. Variable importance in projection (VIP) values ≥ 1.0 were utilized to select characteristic elements. All statistical analyses were conducted using IBM SPSS Statistics 26.0 (IBM Corp., Armonk, NY, USA), and mantel test heatmaps were generated using the online platform for data analysis and visualization (https://www.bioinformatics.com.cn, 30 July 2026). Statistical significance was established at *p* < 0.05.

## 5. Conclusions

Systematic differences in elemental profiles were observed between LJ and LFS, with Fe, Mn, and Na identified as major contributors to species discrimination. Among these elements, Fe and Mn exhibited the highest discriminatory power, and the Fe/Mn ratio demonstrated potential as a complementary chemical indicator for distinguishing LJ from LFS. These findings indicate that elemental profiling can provide valuable chemical information for the authentication and quality evaluation of closely related Lonicera medicinal materials. The network pharmacology and docking analyses yielded in silico preliminary hypotheses regarding the potential functional relevance of Fe and Mn; however, their biological and pharmacological implications must be confirmed through direct experimental investigations. Overall, this work offers elemental fingerprint reference data for distinguishing LJ from LFS, as well as preliminary computational insights for subsequent experimental research exploring the biological effects associated with these elements.

## Figures and Tables

**Table 1:** Validation parameters for the determination of inorganic elements in LJ and LFS using ICP-MS and ICP-AES.

Element	Linear Regression Equation	*R* ^2^	LOD (μg·L^−1^)	LOQ (μg·L^−1^)	Precision RSD (%)	Repeatability RSD (%)	Recovery (%, Mean ± RSD)
Fe	y = 1.2217x − 7.2655	0.9999	0.074	0.2468	0.51	2.14	97.88 ± 1.85, 93.26 ± 2.2, 95.27 ± 1.93
Cu	y = 2.8512x + 0.0218	1	0.052	0.1732	0.51	3.87	96.27 ± 3.18, 94.91 ± 2.99, 100.34 ± 1.05
Mn	y = 3.148x − 21.763	0.9991	0.0341	0.1136	0.68	5.76	95.63 ± 4.75, 96.92 ± 7.57, 94.38 ± 1.61
Zn	y = 1.9916x − 3.6225	1	0.0441	0.1471	0.63	5.2	104.4 ± 2.44, 95.6 ± 2.84, 96.29 ± 2.03
Na	y = 0.852x − 19.869	0.9994	0.0747	0.249	0.53	2.81	99.62 ± 4.46, 95.81 ± 1.04, 94.74 ± 3.76
Cr	y = 4.5413x − 0.0348	1	0.0016	0.0052	0.72	2.08	98.32 ± 7.47, 93.08 ± 1.01, 94.62 ± 1
Mo	y = 3.8518x − 0.0704	1	0.0016	0.0052	1	5.6	96.72 ± 3.65, 95.67 ± 1.03, 96.59 ± 0.95
Ni	y = 5.2139x − 1.9802	0.9996	0.0016	0.0052	0.66	4.42	101.32 ± 2.75, 91.67 ± 1.3, 92.86 ± 1.14
As	y = 1.8572x − 0.0751	1	0.0016	0.0052	1.39	5.25	103.33 ± 4.25, 96.46 ± 3.8, 94.17 ± 2.9
Cd	y = 6.2503x − 0.5702	0.9997	0.0002	0.0005	1.39	5.21	98.00 ± 2.04, 99 ± 1.01, 103.33 ± 2.33

**Table 2:** Molecular docking results of iron and manganese with core target proteins identified through network pharmacology, displaying PDB identifiers, simulated docking scores (kcal/mol), active site residues, docking center coordinates (x, y, z), and protein pocket dimensions (x, y, z).

Protein	PDB ID	Targeting Small Molecules (PubChem CID)	Docking Scores (kcal/mol)	Binding Site Residues	Docking Center (x, y, z)	Protein Pocket Size (x, y, z)
TFRC	6OKD	23925	−1.3	SER-8, SER-536, ALA-7	122.68, −18.47, 33.41	93.0, 10.75, 83.25
FTH1	9LBT	23925	−1.4	GLU-61, GLU-64, HIS-65	10.19, 38.08, 28.86	54.0, 50.25, 58.5
HBA1	7JXZ	23925	−1.3	SER-84, SER-138, VAL-135, ALA-88, THR-137, LEU-136	−13.03, 4.30, 10.61	63.75, 66.75, 73.5
CP	4ENZ	23925	−1.7	ASP-127, ASP-128, LYS-109, GLN-124	6.28, 85.02, 13.53	86.25, 91.5, 90.0
DPP4	6B1E	23925	−1.5	VAL-546, ASP-545, TRP-627, GLY-628	17.60, 57.34, 35.09	132.75, 80.25, 93.75
ALB	8K1Y	23930	−1.2	HIS-105, LYS-106, LEU-103, GLN-104	28.36, −0.33, 18.23	90.0, 53.25, 94.5
ACO1	5TCV	23930	−1.5	SER-212, GLU-35, MET-209, PRO-208	11.16, 16.10, 149.46	50.25, 74.25, 63.0
SOD2	9BWR	23930	−1.2	ASN-142, PRO-141, CYS-140, GLY-122, TRP-123, GLN-143	30.70, 28.43, 89.85	81.75, 63.75, 49.5
SLC39A14	Q15043	23930	−1.1	SER-229, ALA-182, MET-225, LEU-186	−10.49, 16.15, −0.26	98.25, 83.25, 93.0
CP	4ENZ	23930	−1.7	ASP-127, ASP-128, GLN-124, LYS-109	6.22, 84.69, 11.86	86.25, 91.5, 87.75

## Data Availability

The original contributions presented in this study are included in the article/Supplementary Materials. Further inquiries can be directed to the corresponding authors.

## Supplementary Materials

The following supporting information can be downloaded at: https://www.mdpi.com/article/10.3390/ijms27167276/s1.

## Abbreviations

The following abbreviations are used in this manuscript:

LJ	*Lonicerae japonicae* flos
LFS	*Lonicerae* flos
LM	*L. macranthoides*
LH	*L. hypoglauca*
LC	*L. confusa*
LF	*L. fulvotomentosa*
TCM	Traditional Chinese medicine
ICP-MS	inductively coupled plasma mass spectrometry
ICP-AES	inductively coupled plasma atomic emission spectrometry
OPLS-DA	orthogonal partial least squares-discriminant analysis
GO	Gene Ontology
KEGG	Kyoto Encyclopedia of Genes and Genomes
BP	biological process
CC	cellular component
MF	molecular function
FTH1	ferritin heavy chain 1
TFRC	transferrin receptor 1
HBA1	hemoglobin subunit alpha 1
CP	ceruloplasmin
DPP4	dipeptidyl peptidase 4
ALB	albumin
ACO1	aconitase 1
SOD2	superoxide dismutase 2
SLC39A14	solute carrier family 39 member 14
