# Supplementary material for: Comparative Elemental Profiling and Differentiation Analysis of Lonicerae japonicae Flos and Lonicerae Flos Based on ICP-MS/AES and Chemometrics
Source: Int J Mol Sci. 2026 Aug 14;27(16):7276. doi: 10.3390/ijms27167276 (PMC13513504; doi:10.3390/ijms27167276)
Supplement: Supplementary file 1 [file ijms-27-07276-s001.zip › ijms-4456132-supplementary.pdf]

Divergence in the Material Basis of *Lonicerae Japonicae* Flos and *Lonicerae* Flos: Insights from Inorganic Elements and Molecular Docking

Chao Yu[1, 2, †], Yanxia Shu[1, 2, †], Xiuli Li[3], Zongshuo Li[4], Fan Su[3], Meng Sun[3], Zhenghui Liu[3, *], Weidong Li[1, 2, *]

[1] School of Chinese Materia Medica, Beijing University of Chinese Medicine, Beijing 102488, P. R. China; yuchao202111@163.com (C. Y); shuyanxia66@163.com (Y. S)

[2] Collaborative Innovation Center of Aerospace and Traditional Chinese Medicine, Beijing University of Chinese Medicine, Beijing 102488, P. R. China

[3] Tianjin Academy of Agricultural Sciences, Tianjin 300381, P. R. China; lixiuli@taas.ac.cn (X. L); sufan@taas.ac.cn (F. S); axsuar@163.com (M. S)

[4] School of Life Sciences, Beijing University of Chinese Medicine, Beijing 102488, P. R. China; lizongshuo2009@126.com (Z. L)

[*] Correspondence: liweidong2005@126.com (W. L), liuzhenghui@taas.ac.cn (Z. L)

[†] These authors contributed equally to this work.

Table S1 Summary of tested samples.

Species	Sample no.	Origin
Lonicerae Japonicae	1–7	Pingyi County, Shandong Province
Flos(LJF)	8–13	Fengqiu County, Henan Province
	14–20	Julu County, Hebei Province
	21–31	Longhui County, Hunan Province
L. macranthoides (LM)	32–35	Xupu County, Hunan Province
	36–50	Xiushan County, Chongqing Municipality
L. hypoglauca (LH)	51–60	Xincheng County, Guangxi Province
	61–66	Mashan County, Guangxi Province
L. confusa (LC)	67–70	Guangzhou City, Guangdong Province
	71–72	Nanxiong City, Guangdong Province
	73–76	Ruyuan County, Guangdong Province
	77–78	Qujiang County, Guangdong Province
	79	Xinfeng County, Guangdong Province
	80–81	Shaoguan City, Guangdong Province
L. fulvotomentosa (LF)	82–97	Anlong County, Guizhou Province

Table S2 Operating parameters for microwave digestion of trace elements extracts.

Step	Temperature (°C)	Power (W)	Temperature increasing speed ($°C·min^{-1}$)	Duration (min)
1	120	1600	9	1
2	160	1600	8	5
3	185	1600	8	25

Table S3 Levels of trace elements in *L. Japonicae* Flos and *L.* Flos (n = 3).

No.	Concentration ($\mu g \cdot g^{-1}$)									
	Fe	Cu	Mn	Zn	Na	Cr	Mo	Ni	As	Cd
					**LJF**					
1	164.3	3.4	14.6	12.4	55.1	1.57	1.946	1.329	0.113	0.077
2	112.8	2.7	18.9	12.3	33.5	1.80	11.969	1.156	0.026	0.062
3	77.8	3.1	23.0	11.5	31.0	1.60	3.242	2.888	0.069	0.091
4	59.2	3.6	21.1	13.0	34.1	1.39	2.502	3.379	0.048	0.164
5	59.3	2.9	10.1	12.0	37.7	1.41	0.725	0.892	0.047	0.026
6	144.5	3.1	17.9	13.8	40.4	1.35	2.029	2.390	0.033	0.110
7	34.4	2.1	7.8	9.2	31.5	1.64	13.051	2.479	0.059	0.094
8	43.8	4.1	25.3	15.4	43.4	1.51	1.112	0.579	0.087	0.039
9	48.7	3.6	8.2	10.8	35.1	1.59	1.226	0.881	0.112	0.021
10	48.2	3.2	6.4	13.8	27.3	1.46	0.547	0.602	0.075	0.023
11	79.5	3.8	11.3	14.7	42.7	1.40	1.356	1.131	0.061	0.016
12	61.8	3.5	11.2	11.4	38.4	1.84	2.931	1.509	0.150	0.023
13	46.3	3.7	10.7	9.6	37.3	1.70	1.010	1.021	0.045	0.021
14	70.9	3.7	11.8	10.2	46.1	1.61	1.166	0.882	0.109	0.026
15	74.8	3.6	10.0	11.7	38.1	1.54	1.317	0.422	0.050	0.016
16	104.0	3.3	9.4	12.1	46.0	1.63	1.150	0.540	0.056	0.022
17	135.4	3.2	12.1	11.0	81.2	1.54	1.641	1.185	0.116	0.038
18	58.2	3.2	11.6	9.8	32.0	2.06	0.588	0.767	0.046	0.030
19	62.1	3.3	10.7	12.7	41.3	1.63	0.884	0.523	0.101	0.030
20	58.7	4.0	12.2	12.2	38.5	1.54	0.421	0.568	0.129	0.034
					**LM**					
21	28.3	4.2	20.5	27.5	38.8	1.18	0.281	0.323	0.022	0.217
22	17.0	3.2	58.7	10.3	23.0	1.51	1.412	0.638	0.039	0.443
23	21.0	3.5	43.2	14.2	24.4	1.47	0.594	0.614	0.081	0.287
24	22.0	4.2	42.0	14.3	30.1	1.28	1.325	0.566	0.059	0.342
25	24.7	3.9	63.0	14.5	29.3	1.09	0.570	0.523	0.100	0.312
26	21.7	3.8	66.2	17.2	33.1	1.53	0.675	0.637	0.127	0.399
27	20.9	3.3	52.1	11.3	23.8	1.29	1.031	0.506	0.074	0.318
28	28.4	3.6	44.5	9.3	23.5	1.68	0.552	0.587	0.057	0.332
29	42.5	4.1	45.4	13.4	55.9	1.42	0.462	0.507	0.061	0.355
30	39.4	3.5	42.3	19.3	31.0	1.59	0.722	0.743	0.065	0.289
31	24.1	4.1	71.7	15.0	34.3	1.63	0.768	0.731	0.147	0.534
32	37.1	4.1	38.7	12.1	26.8	1.27	1.620	0.309	0.018	0.159
33	33.5	4.4	56.7	13.7	23.3	1.49	1.021	0.668	0.035	0.389
34	29.7	3.8	62.2	14.9	27.1	1.21	0.135	0.468	0.033	0.294
35	28.3	3.4	51.2	13.5	26.8	1.56	0.673	0.574	0.055	0.262
36	41.5	3.8	57.2	16.9	35.9	1.56	0.235	1.166	0.081	0.657

37	36.0	3.5	56.1	19.4	46.1	1.58	0.426	0.966	0.073	0.606
38	28.9	3.6	102.1	12.5	39.8	1.58	0.448	0.934	0.026	0.680
39	36.6	3.6	91.2	13.7	33.7	2.67	0.789	1.148	0.335	0.591
40	25.2	3.7	46.5	15.5	34.9	1.99	0.666	0.645	0.042	0.316
41	52.9	3.9	60.1	12.9	33.6	1.58	0.301	1.002	0.057	0.540
42	48.5	3.5	49.8	22.9	36.1	1.53	0.959	0.834	0.084	0.599
43	29.5	3.8	64.7	13.7	31.3	2.88	1.063	0.840	0.108	0.561
44	35.9	3.7	55.6	16.5	33.7	1.72	1.592	0.728	0.123	0.313
45	18.3	3.7	48.1	13.5	27.0	1.65	0.794	0.642	0.018	0.414
46	23.1	3.8	35.3	23.9	42.4	1.46	0.281	0.645	0.043	0.265
47	18.6	3.2	52.3	13.2	27.6	1.40	0.540	0.567	0.102	0.503
48	44.9	4.1	74.7	13.9	34.9	1.50	1.581	1.174	0.135	0.753
49	86.0	4.1	64.8	13.8	31.7	1.87	0.790	0.930	0.077	0.692
50	20.5	3.2	72.4	12.3	28.4	1.10	3.785	2.018	0.008	0.613
					**LH**					
51	62.2	3.9	27.3	10.0	32.0	1.72	1.336	0.993	0.378	0.175
52	52.0	4.0	26.1	16.4	36.5	1.54	1.095	0.632	0.085	0.159
53	48.2	4.1	21.0	14.7	44.9	1.56	0.694	0.494	0.057	0.201
54	49.8	4.4	18.0	10.3	29.3	1.53	0.864	0.623	0.073	0.222
55	42.0	4.8	14.5	10.2	30.1	1.44	0.849	0.655	0.111	0.198
56	40.1	4.6	13.9	12.0	32.2	1.59	0.770	0.746	0.066	0.180
57	23.0	4.9	9.2	10.2	29.0	1.25	0.806	0.446	0.036	0.029
58	22.3	4.5	10.3	11.3	25.3	1.41	1.474	0.634	0.284	0.081
59	36.8	4.4	10.8	13.9	25.5	1.31	0.878	0.511	0.083	0.155
60	43.1	4.7	24.3	15.0	34.0	1.38	0.759	0.754	0.038	0.158
61	33.0	4.4	12.1	11.3	28.7	1.82	0.735	0.496	0.123	0.126
62	27.1	3.8	11.2	7.0	24.6	1.90	1.404	0.442	0.187	0.130
63	65.6	5.1	11.9	16.8	36.1	1.53	0.395	0.541	0.080	0.212
64	55.0	4.6	10.9	13.8	33.0	1.49	1.026	0.487	0.067	0.176
65	37.8	4.6	9.1	13.4	29.7	1.37	0.669	0.416	0.056	0.141
66	29.6	5.1	9.6	15.5	34.2	1.40	0.802	0.567	0.082	0.192
					**LC**					
67	19.5	3.5	51.5	12.3	72.1	0.15	0.081	0.067	0.005	0.020
68	19.7	3.0	62.7	11.9	22.0	0.18	0.147	0.066	0.004	0.031
69	22.5	3.2	52.8	12.1	26.4	0.14	0.064	0.090	0.014	0.007
70	32.9	4.0	68.0	11.5	34.2	0.15	0.050	0.144	0.005	0.027
71	115.1	4.2	46.1	11.9	27.6	0.17	0.051	0.160	0.010	0.042
72	34.4	3.9	59.1	11.5	28.5	0.18	0.032	0.076	0.004	0.039
73	23.6	4.1	95.7	21.7	31.4	0.17	0.117	0.085	0.005	0.054
74	22.7	3.5	57.4	10.4	25.5	0.15	0.083	0.074	0.007	0.034
75	18.7	3.6	57.0	11.9	26.5	0.14	0.102	0.060	0.010	0.038

76	39.4	3.9	26.5	13.5	33.7	0.16	0.243	0.084	0.015	0.003
77	19.7	3.6	65.0	10.3	27.4	0.15	0.067	0.071	0.003	0.036
78	49.2	3.7	83.3	11.5	24.4	0.14	0.022	0.079	0.005	0.030
79	71.9	3.8	45.7	9.9	43.9	0.17	0.098	0.102	0.034	0.030
80	39.6	4.1	50.4	12.1	30.9	0.18	0.081	0.065	0.003	0.026
81	31.3	3.8	70.2	9.8	27.4	0.13	0.071	0.111	0.007	0.042
**LF**										
82	38.3	3.5	35.0	8.2	23.0	0.17	0.116	0.049	0.014	0.006
83	50.9	3.6	14.2	11.1	29.2	0.16	0.126	0.045	0.008	0.006
84	33.3	4.1	11.6	12.5	39.0	0.13	0.042	0.045	0.005	0.010
85	35.5	5.0	13.8	12.6	38.7	0.15	0.106	0.056	0.012	0.012
86	15.2	4.2	10.2	6.8	31.4	0.12	0.032	0.031	0.003	0.007
87	17.5	4.3	9.2	9.2	34.3	0.17	0.146	0.056	0.015	0.005
88	25.1	4.0	10.6	7.7	28.6	0.17	0.118	0.035	0.009	0.008
89	31.0	3.7	12.0	8.0	27.3	0.15	0.124	0.046	0.008	0.005
90	27.3	3.7	15.4	8.8	26.9	0.16	0.107	0.043	0.006	0.009
91	36.4	4.2	17.8	7.3	24.6	0.15	0.139	0.048	0.004	0.012
92	39.1	4.6	18.2	8.7	25.7	0.15	0.094	0.043	0.007	0.008
93	43.0	5.2	17.1	12.8	36.6	0.13	0.134	0.049	0.011	0.009
94	40.6	3.9	26.8	9.7	21.9	0.15	0.047	0.057	0.005	0.002
95	41.0	3.2	8.1	9.2	25.2	0.16	0.104	0.053	0.010	0.003
96	25.5	3.9	13.5	9.9	27.0	0.15	0.075	0.053	0.006	0.008
97	17.7	3.3	11.5	8.5	25.5	0.26	0.102	0.165	0.010	0.009

Table S4 The mean of trace element content in *L. Japonicae* Flos and *L.* Flos.

Species	Content, x ± s ($\mu g \cdot g^{-1}$)				
	LJF	LM	LH	LC	LF
Fe	77.235±36.21a	32.17±13.90b	41.73±13.13b	37.35±25.87b	32.34±32.34b
Cu	3.36±0.46c	3.74±0.32b	4.49±0.39a	3.73±0.34b	4.03±0.56b
Mn	13.22±5.25b	56.31±16.21a	15.01±6.29b	59.43±16.40a	15.31±6.93b
Zn	11. 98±1.65b	15.17±3.98a	12.61±2.75b	12.15±2.82b	9.44±1.89c
Na	40.54±11.50a	32.28±7.31b	31.57±5.09b	32.13±12.23b	29.06±5.42b
Cr	1.59±0.17a	1.58±0.39a	1. 52±0.18a	0.16±0.02b	0.16±0.03b
Mo	2.54±3.50a	0.87±0.69b	0.91±0.29b	0.09±0.05b	0.1±0.03b
Ni	1. 26±0.85a	0.75±0.33b	0.59±0.15b	0.09±0.03c	0.05±0.03c
As	0.08±0.04a	0.08±0.06a	0.11±0.09a	0.01±0.01b	0.01±0.00b
Cd	0.05±0.04c	0.43±0.16a	0.16±0.05b	0.03±0.01c	0.01±0.00c

Note: Peers labeled with different letters indicate significant differences ($P < 0.05$).
